# *In vitro* Effects of Antimicrobial Agents on Planktonic and Biofilm Forms of *Staphylococcus saprophyticus* Isolated From Patients With Urinary Tract Infections

**DOI:** 10.3389/fmicb.2019.00040

**Published:** 2019-01-28

**Authors:** Katheryne Benini Martins, Adriano Martison Ferreira, Valéria Cataneli Pereira, Luiza Pinheiro, Adilson de Oliveira, Maria de Lourdes Ribeiro de Souza da Cunha

**Affiliations:** ^1^Department of Microbiology and Immunology, Institute of Biosciences, UNESP – Universidade Estadual Paulista, Botucatu, Brazil; ^2^Department of Tropical Diseases, Botucatu School of Medicine University Hospital, UNESP – Universidade Estadual Paulista, Botucatu, Brazil

**Keywords:** *Staphylococcus saprophyticus*, biofilm, antimicrobial resistance, MICB, MBCB

## Abstract

Bacterial biofilms play an important role in urinary tract infections (UTIs), being responsible for persistent infections that lead to recurrences and relapses. *Staphylococcus saprophyticus* is one of the main etiological agents of UTIs, however, little is known about biofilm production in this species and especially about its response to the antimicrobial agents used to treat UTIs when a biofilm is present. For this reason, the aim of this work was to evaluate the response of *S. saprophyticus* biofilms to five antimicrobial agents. *Staphylococcus saprophyticus* was evaluated for antimicrobial susceptibility in its planktonic form by means of minimum inhibitory concentration (MIC) and in biofilms by means of minimum inhibitory concentration in biofilm (MICB) against the following antimicrobial agents by the microdilution technique: vancomycin, oxacillin, trimethoprim/sulfamethoxazole, ciprofloxacin, and norfloxacin. Of the 169 *S. saprophyticus* studied, 119 produced a biofilm as demonstrated by the polystyrene plate adherence method. Biofilm cells of *S. saprophyticus* exhibited a considerable increase in MICB when compared to the planktonic forms, with an increase of more than 32 times in the MICB of some drugs. Some isolates switched from the category of susceptible in the planktonic condition to resistant in the biofilm state. Statistical analysis of the results showed a significant increase in MICB (*p* < 0.0001) for all five drugs tested in the biofilm state compared to the planktonic form. Regarding determination of the minimum bactericidal concentration in biofilm (MBCB), there were isolates for which the minimum bactericidal concentration of all drugs was equal to or higher than the highest concentration tested.

## Introduction

In order to survive in hostile environments such as in host tissues (antibodies, phagocytes, etc.,) or on an inert surface where they are exposed to inhospitable conditions (UV light, desiccation, heat, cold), bacteria adapt by forming adherent populations (sessile bacteria) organized in a structure called biofilm (Mah and O’Toole, 2001).

Li et al. (2005) demonstrated that biofilm formation in *Staphylococcus* spp. depends on Polysaccharide Intercellular Adhesin (PIA), whose biosynthesis is mediated by the *ica* operon. This operon contains the *ica*ADBC genes and the regulatory *ica*R gene, which is transcribed in the direction opposite to the *ica* operon. In the case of the *ica*R gene, some studies have suggested that its product is a transcription repressor that plays an adaptive role in the regulation of the expression of the *ica* operon according to environmental conditions. Some factors such as anaerobic growth, the presence of antibiotics at subinhibitory concentrations, and environmental stress such as high osmolarity may increase expression of the *ica* operon. In addition to PIA, the existence of *ica*-independent mechanisms for biofilm formation in *Staphylococcus* spp., such as proteins and DNA, has been highlighted (Mendoza-Olazarán et al., 2015).

Once formed, these biofilms render the cells less accessible to the defense system of the organism, impairing the action of antibiotics. Biofilms thus represent basic survival strategies of these microorganisms, a fact that explains why biofilms are considered to be of major public health importance. Furthermore, the proximity of cells inside microcolonies or between microcolonies provides an excellent environment for the exchange of genetic material. The mechanism of conjugation, i.e., the transfer of plasmids between bacteria, occurs at a higher proportion between bacterial cells in biofilms than between planktonic cells (Águila-Arcos et al., 2017).

In the laboratory, the effectiveness of an antibiotic is evaluated with the microorganism in its planktonic form (free cells). However, these assays only reveal the concentration of the chemotherapeutic agent that is necessary to inhibit growth or kill planktonic bacteria (Jorgensen and Ferraro, 2009). Maximum resistance to antibiotics is achieved once microorganisms complete the formation of the mature biofilm (Høiby et al., 2010). For some antibiotics, the concentration required to kill sessile bacteria can be up to a thousand times greater than the concentration required to kill exactly the same strain in its planktonic form (Nickel et al., 1985; Aslam, 2008). Therefore, in some circumstances, the use of planktonic bacteria for the selection of chemotherapeutic agents may be inappropriate.

Biofilm formation can be considered a virulence determinant that is responsible for the long-term persistence of bacteria in the genitourinary tract (Costerton et al., 1999). Urinary catheters and other prosthetic devices predispose to urinary tract infections (UTIs) by destroying natural barriers (urethral sphincter) and providing a nidus for infection that serves as a substrate for biofilm formation. Bacterial biofilms play an important role in UTIs, being responsible for persistent infections that lead to recurrences and relapses (Delcaru et al., 2016).

The most commonly prescribed antibiotics for the treatment of UTIs are trimethoprim/sulfamethoxazole, fluoroquinolones, first- and second-generation cephalosporins, amoxicillin + clavulanate, and nitrofurantoin (Lee et al., 2008). According to the CLSI M100-S26 document (2016), routine susceptibility testing of urinary *S. saprophyticus* isolates is not recommended since this microorganism is normally susceptible to the antimicrobial agents used to treat acute uncomplicated UTIs (nitrofurantoin, sulfamethoxazole/trimethoprim, or a fluoroquinolone). However, 17.6% of the *S. saprophyticus* isolated from UTIs tested by Ferreira et al. (2012) were resistant to sulfamethoxazole/trimethoprim, a fact that may lead to therapeutic failure when UTIs are treated empirically. Antibiotic resistance seems to have emerged also among *S. saprophyticus* strains and antimicrobial susceptibility testing of these strains is therefore necessary.

*Staphylococcus saprophyticus* is one of the main etiological agents of UTIs, however, little is known about biofilm production in this species and especially about its response to the antimicrobial agents used to treat UTIs when a biofilm is present. For this reason, the aim of this work was to evaluate the response of *S. saprophyticus* biofilms to five antimicrobial agents.

## Materials and Methods

### Samples

*Staphylococcus saprophyticus* isolated from the urine of different patients were used in the study. The strains were obtained in a prospective study through isolation in the Laboratory of Microbiology, University Hospital of the Botucatu School of Medicine (HC-FMB), SP, Brazil, in 2013 and 2014 or were obtained from a culture collection established in 2008. The samples were collected from patients originating from wards, outpatient clinics, emergency rooms, and basic health units of Botucatu and region. The present study was approved by the institutional Ethics Committee (Protocol 16269813.1.0000.5411) and was exempt from the requirement of free informed consent of the participants in this study since we did not use clinical data of the patients and had no contact with the patients. Bacteria had previously been isolated from the patients and were stored at the Laboratory of Microbiology (HC-FMB).

Individuals of both genders and all ages with *S. saprophyticus*-positive urine cultures compatible with UTI, with a colony count equal to or greater than 100,000 colony forming units per milliliter of urine ( ≥ 10^5^ CFU/mL) according to the criteria of Kass (1956), were included. Samples were collected according to the urine collection protocol of the service.

The isolates were seeded on blood agar with 5% sheep blood (secondary isolation) and stained by the Gram staining method for the assessment of purity and observation of their specific morphology and staining. After confirmation of these characteristics, the strains were submitted to the catalase, DNAse, and tube coagulase (gold standard) tests to distinguish *Staphylococcus aureus* and coagulase-negative staphylococci (CoNS) as recommended by Koneman et al. (1997).

### DNA Extraction and Identification of *S. saprophyticus*

DNA was extracted from isolates identified as CoNS with the Illustra^®^Kit (GE Healthcare) according to manufacturer’s instructions.

Isolates identified as CoNS were genotyped using primers targeting conserved sequences adjacent to the 16S and 23S genes by the internal transcribed spacer-PCR (ITS-PCR) technique described by Couto et al. (2001). The G1 “GAAGTCGTAACAAGG” 16S and L1 “CAAGGCATCCACCGT” 23S primers were used. The efficiency of the amplifications was monitored by electrophoresis on 3% MetaPhor agarose prepared in 1X TBE buffer and stained with SYBR Safe. The following international reference strains were used as controls: *S. epidermidis* (ATCC 12228), *S. epidermidis* (ATCC 35983), *S. haemolyticus* (ATCC 29970), *S. hominis* (ATCC 27844), *S. hominis* subsp. *novobiosepticus* (ATCC 700237), *S. lugdunensis* (ATCC 700328), *S. saprophyticus* (ATCC 15305), and *S. warneri* (ATCC 10209).

### Detection of *mecA* Gene for Oxacillin Resistance

For detection of the *mecA* gene, PCR was performed using the *mecA1* (AAA ATC GAT GGT AAA GGT TGG) and *mecA2* (AGT TCT GCA GTA CCG GAT TTG) – 533 (bp) primers according to the parameters described by Murakami et al. (1991). International reference strains were included in all reactions: *S. aureus* ATCC 33591 (positive) and *S. aureus* ATCC 25923 (negative).

Agarose gels were prepared at a concentration of 2% in 1X TBE, stained with SYBR Safe DNA Gel Stain^®^ (Invitrogen), and visualized under a UV transilluminator.

### Detection of Biofilm Production by the Polystyrene Plate Adherence Method (Christensen et al., 1985) Modified by Oliveira and Cunha (2010)

The method of detecting biofilm production in culture plates proposed by Christensen et al. (1985) was used, with modifications proposed by Oliveira and Cunha (2010). This method is based on the spectrophotometric reading of optical density of the adherent material produced by the bacteria. International reference strains used as positive (*S. aureus* ATCC 29213, *S. epidermidis* ATCC 35983) and negative controls (*S. aureus* ATCC 33591, *S. epidermidis* ATCC12228) and sterile TSB were included in all tests. Optical density reading was carried out in an ELISA reader (Labsystems, model Multiskan EX) using a 540-nm filter. Samples were classified as negative when the cut-off value corresponded to the classification of non-adherent ( ≤ 0.111) and as positive when the cut-off value corresponded to the classification of weakly adherent (>0.111 or ≤0.222) or strongly adherent (>0.222). These cut-offs values were established by Oliveira and Cunha (2010).

### Evaluation of Biofilm Formation With Visualization by Scanning Electron Microscopy (SEM) in an Isolate of Biofilm-Producing *S. saprophyticus*

A biofilm-producing *S. saprophyticus* isolate in the polystyrene plate adherence test was selected for confirmation of biofilm production by SEM. The biofilm-producing strain was first isolated in BHI broth and 10^8^ CFU of bacteria were transferred to a conical tube (Falcon-CORNING) containing 2 mL TSB culture medium prepared with 2% glucose and a 0.5-cm segment of VYGON umbilical catheter (reference 1270.04, 0.8 mm × 1.5 mm diameter). The tube was incubated under constant stirring for 48 h at 100 rpm/37°C for bacterial growth and biofilm formation. After this period, the catheter segment was removed, washed with PBS, immersed in 2.5% glutaraldehyde solution, fixed in an increasing alcohol series (15, 30, 50, 70, 90, and 100%) for 15 min each, dried in a vacuum centrifuge for 5 min, metallized with gold, and visualized under a scanning electron microscope to evidence biofilm production.

### Determination of MIC of Vancomycin, Oxacillin, Norfloxacin, Ciprofloxacin, and Trimethoprim/Sulfamethoxazole for Planktonic Cells of *S. saprophyticus* by the Broth Microdilution Method

The broth microdilution method was used for determination of the (MIC) for planktonic cells of *S. saprophyticus*. Sterile microtiterplates with Müller-Hinton broth adjusted with cations (Oxoid, United Kingdom) as recommended by the CLSI (2016) were used. A stock solution of each drug was prepared in 3,200 μg/mL distilled water. Serial dilutions were made in a microtiter plate containing Müller-Hinton broth at concentrations on a logarithmic scale of two, comprising the breakpoints (CLSI, 2016), in a final volume of 100 μL. For preparation of the inoculum, the isolates were first seeded on blood agar. After incubation for 24 h, isolated colonies were seeded in BHI broth and the bacterial suspensions were adjusted to a turbidity of 0.5 McFarland standard (1 × 10^8^ CFU/mL), diluted at 1:1000, and added to the wells in a final volume of 200 μL and final bacterial concentration of 5× 10^4^ CFU/well. The plates were incubated in an oven at 35°C and the MIC was read after 24 and 48 h of incubation. A positive control containing the broth and bacterial suspension and a negative control containing only the Müller-Hinton broth were used. In addition, *Enterococcus faecalis* ATCC 29212 and *S. aureus* ATCC 29213 (susceptible to vancomycin) were used as negative controls, and *E. faecalis* ATCC 51299 (resistant to vancomycin) and *S. aureus* ATCC 33591 (resistant to oxacillin) were used as positive controls. The MIC was defined as the lowest concentration of antimicrobial that completely inhibited the growth of the microorganism as detected by the naked eye. Wells with turbidity and/or the presence of bacteria at the bottom of the well were classified as positive growth. The susceptibility and resistance cut-offs recommended by the CLSI (2016) were used to determine the MIC for planktonic cells. The same cut-offs were used to evaluate the biofilm antimicrobial susceptibility of the isolates since no standards exist for biofilm tests.

### Determination of (MICB) and (MBCB) of Vancomycin, Oxacillin, Norfloxacin, Ciprofloxacin, and Trimethoprim/Sulfamethoxazole for *S. saprophyticus* Biofilm by the Broth Microdilution Method

Bactericidal concentrations for biofilms (MBCB) were determined by adapting the test method described by Frank et al. (2007). The isolates cultured for 22 h in TSB with 2% glucose were adjusted to a turbidity of 1.0 McFarland standard (corresponding to 1 × 10^8^ to 2 × 10^8^ CFU/mL) and diluted at 1:50 in TSB with 2% glucose. Aliquots (200 μL) were plated in 96-well flat bottom plates (Nuclon Delta, Nunc, Denmark), covered with a 96-pin cap (Nunc-TSP; Nunc), and incubated for 24 h to allow biofilm formation on the pins. To remove non-adherent cells, the biofilms formed on the pins were washed by immersion in a series of three 96-well plates filled with 200 μL of sterile saline phosphate-buffered saline (PBS). The cap with the pins was placed on a flat bottom plate prepared for broth microdilution susceptibility testing. The wells contained 200 μL of antimicrobial agent diluted in CAMHB (Müller-Hinton broth supplemented with cations, 100 mg/mL calcium, and 50 mg/mL magnesium) or 200 μL of CAMHB without drugs as positive growth control. The biofilms were exposed to the antimicrobials for 24 h. The cap with the pins was removed, washed three times in PBS as described above, and transferred to 96-well plates containing 200 μL TSB plus 2% glucose. On that occasion, prior to discarding the plate with the antibiotics, a “naked eye” reading was performed to determine the MIC of the antibiotics for biofilm cells (MICB). Subsequently, the biofilm cells formed on the cap pins were dislodged by sonication for 5 min at 40 kHz (Hielscher, Ultrasonic Technology, UIP250MTP) in 96-well plates containing fresh culture medium for cell recovery. The cap with the pins was discarded and replaced with a normal cap and optical density was measured in a plate reader equipped with a 600-nm filter. Wells containing TSB plus 2% pure glucose (without inoculation) were used as spectrophotometric sterility controls. The plate was incubated for 24 h and a second optical density measurement at 600 nm was taken. The MBCB was defined as the lowest concentration of the drug that exhibited a change in optical density at 600 nm of 10% of the reading obtained for the positive growth control between the readings performed before incubation and after 24 h. For better control of the efficacy of the test, we used the biofilm-producing strain *S. epidermidis* ATCC 35983 and the non-producing strain *S. epidermidis* ATCC 12228 as controls.

### Statistical Analysis

Correlation analysis between antimicrobial susceptibility and the inhibitory concentration of the drugs for planktonic and biofilm bacteria was performed using the Chi-squared test or Fisher’s exact test (SPSS^®^ 13.0 software), adopting a level of significance <0.05.

## Results

### Detection of Biofilm Production by the Polystyrene Plate Adherence Method

A total of 169 samples of *S. saprophyticus* isolated from patients with UTI were used. Of these, 119 (70.4%) produced a biofilm and 88 (52.1%) were classified as strongly adherent and 31 (18.3%) as weakly adherent.

### Evaluation of Biofilm Formation With Visualization by SEM

**FIGURE 1:**
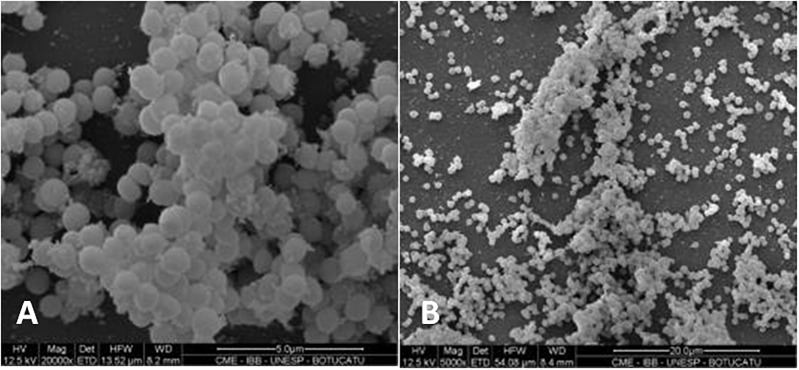
Scanning electron micrograph showing the biofilm structure in *Staphylococcus saprophyticus*. Magnification: **(A)** 20,000×; **(B)** 5,000×.

An *S. saprophyticus* isolate classified as strongly adherent in the evaluation of biofilm production on polystyrene plates was selected for SEM analysis of biofilm production. Figure 1 shows the biofilm structure produced by *S. saprophyticus* isolated from a case of UTI.

### Evaluation of Antimicrobial Susceptibility of Planktonic and Biofilm Cells of *S. saprophyticus*

Biofilm antimicrobial susceptibility was evaluated in the 119 isolates producing biofilms on polystyrene plates. The same drugs as those employed to evaluate antimicrobial susceptibility in planktonic isolates for determination of MIC were used to test the biofilm antimicrobial susceptibility by establishing the (MICB; Table 1).

**Table 1:** Comparison of drug resistance profile between planktonic and biofilm cells of *Staphylococcus saprophyticus*.

	Planktonic bacteria		Bacteria in biofilm	
		
Drug	R (%)	IR (%)	R (%)	IR (%)
Vancomycin	0 (0)	0 (0)	9 (7.6)	19 (16.0)
Oxacillin	117 (98.3)	●	119 (100.0)	●
Norfloxacin	0 (0)	0 (0)	26 (21.8)	15 (12.6)
Ciprofloxacin	0 (0)	0 (0)	24 (20.2)	6 (5.0)
Trim/Sut	21 (17.7)	●	58 (48.7)	●


*●, Drugs without intermediate resistance MIC; R, resistant; IR, intermediate resistance; Trim/Sut, trimethoprim/sulfamethoxazole.*

**FIGURE 2:**
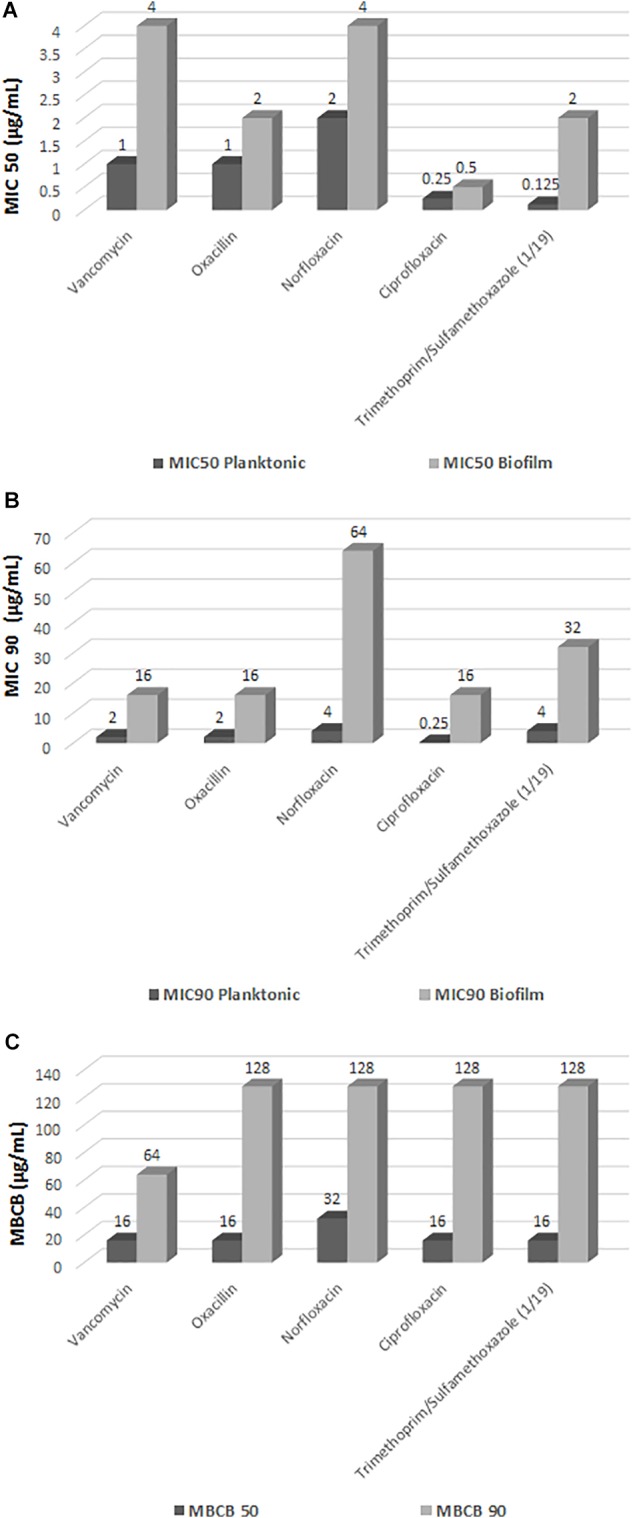
Minimum inhibitory concentration (MIC) in planktonic cells of *Staphylococcus saprophyticus*, minimum inhibitory concentration in biofilm (MICB), and minimum bactericidal concentration in biofilm (MBCB). **(A)** MIC 50 (μg/mL); **(B)** MIC 90 (μg/mL); **(C)** MBCB.

The determination of MIC in planktonic cells against the five antimicrobials revealed that 117 (98.3%) isolates were resistant to oxacillin, with MIC_50_ of 1 μg/mL and MIC_90_ of 2 μg/mL, but only three isolates (2.5%) were positive for the *mecA* gene. These three isolates exhibited the highest MIC (256 μg/mL), while the other 116 showed MIC ranging from ≤ 0.25 to 2 μg/mL. In addition, 21 (17.7%) isolates were resistant to trimethoprim/sulfamethoxazole, with MIC_50_ of 0.25/2.38 μg/mL and MIC_90_ of 4/76 μg/mL. All isolates were susceptible to vancomycin with MIC_50_ of 1 μg/mL and MIC_90_ of 2 μg/mL, to norfloxacin with MIC_50_ of 2 μg/mL and MIC_90_ of 4 μg/mL, and to ciprofloxacin with MIC_50_ and MIC_90_ of 0.25 μg/mL (Figure 2).

Using the criteria for interpretation of susceptibility tests recommended by the CLSI (2016) for determination of MIC in planktonic CoNS as a guideline to evaluate the antimicrobial susceptibility of the biofilm isolates, none of the drugs was found to be totally effective against the biofilm isolates. Statistical analysis of the results showed a significant increase in MICB (*p* < 0.0001) for all five drugs tested in the biofilm state compared to the planktonic forms (Figure 2).

There was a considerable increase in susceptible planktonic isolates that became resistant in the biofilm state (Table 1). Of the 119 biofilm isolates analyzed, 28 (23.5%) exhibited intermediate resistance or resistance to vancomycin (MICB 1 to 64 μg/mL). All isolates were resistant to oxacillin (MICB 0.5 to 2048), 41 (34.4%) exhibited intermediate resistance or resistance to norfloxacin (MICB 2 to 64 μg/mL), 30 (25.2%) demonstrated intermediate resistance or resistance to ciprofloxacin (MICB 0.125 to 64 μg/mL), and 58 (48.7%) were resistant to trimethoprim/sulfamethoxazole (MICB 0.06/1.18 μg/mL to 64/1,216 μg/mL), considering the CLSI (2016) cut-off point for resistance in planktonic cells (Table 1 and Figure 2). Regarding resistance to trimethoprim/sulfamethoxazole, it is important to note that 21 (17.7%) of the 58 (48.7%) isolates resistant to MICB were already resistant in the MIC evaluation of this drug; thus, 37 (31.1%) of the isolates changed from susceptible to resistant in the biofilm state.

The biofilm isolates exhibited a considerable increase in MICB when compared to the planktonic forms, with an increase of more than 32 times in the values of some drugs. Some isolates switched from the category of susceptible in the planktonic condition to resistant in the biofilm state (Figure 2 and Table 2).

**Table 2:** Variation of the increase in MIC and change of the category from susceptible to resistant in relation to planktonic cells and in biofilm.

	2X (%)	4X(%)	8X(%)	16X(%)	32X(%)	64X(%)	128X(%)	256X (%)	S-I(%)	S-R(%)
Vancomycin	59 (49.5)	32 (26.9)	10 (8.4)	9 (7.6)	7 (5.9)	2 (1.7)	–	–	19 (16.0)	9 (7.6)
Oxacillin	71 (59.7)	21 (17.6)	9 (7.6)	8 (6.7)	7 (5.9)	2 (1.7)	1 (0.8)	–	●	2 (1.7)
Norfloxacin	83 (69.7)	10 (8.4)	7 (5.9)	10 (8.4)	9 (7.6)	–	–	–	15 (12.6)	26 (21.8)
Ciprofloxacin	57 (47.9)	27 (22.7)	11 (9.3)	3 (2.5)	5 (4.2)	6 (5.0)	5 (4.2)	5 (4.2)	6 (5.0)	24 (20.2)
Trim/Sut	28 (23.5)	28 (23.5)	14 (11.8)	14 (11.8)	13 (10.9)	7 (5.9)	7 (5.9)	8 (6.7)	●	37 (31.1)


*●, Drugs without intermediate resistance MIC; X, Number of times MIC increased in biofilm samples; S-I, susceptible-intermediate: percentage of isolates with intermediate resistance only in the presence of the biofilm; S-R, susceptible-resistant: percentage of isolates that were resistant only in the presence of the biofilm; Trim/Sut, trimethoprim/sulfamethoxazole.*

**FIGURE 3:**
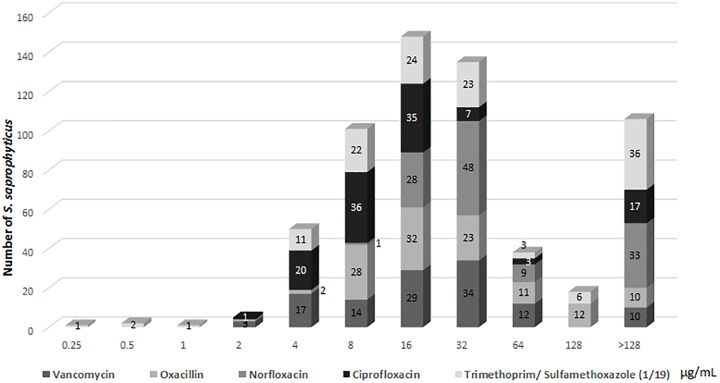
Profile of (MBCB) of *Staphylococcus saprophyticus.*

Regarding determination of the MBCB, there were isolates for which the minimum bactericidal concentration of all drugs was equal to or higher than the highest concentration tested (Figure 3), with emphasis on norfloxacin with 33 (27.7%) samples with MBCB > 128 μg/mL and trimethoprim/sulfamethoxazole with 36 (30.2%) samples with MBCB > 128/2,432 μg/mL.

## Discussion

The formation of bacterial biofilms is the basis of many persistent infectious diseases. This persistence is attributed mainly to the increased antibiotic resistance of biofilm cells (Mah, 2012).

The MIC has been used as a gold standard to determine the antimicrobial susceptibility of pathogenic bacteria (Costerton et al., 1995). When MIC determination reveals that the drug is not effective in inhibiting the growth of a given organism, the drug in question will not be used for the treatment of infection because it will be clinically ineffective (Potera, 1999). However, if a microorganism is considered susceptible *in vitro*, it does not necessarily mean that the drug will have the same effect *in vivo* (Pratt and Kolter, 1999; Mendoza-Olazarán et al., 2015; Algburi et al., 2017). In routine clinical laboratories, antimicrobial susceptibility testing for antibiotic selection continues to be performed using planktonic cells, a fact that impairs evaluation of the efficacy of the antimicrobial tested since these bacteria are protected by the biofilm in the patient and the response will not be the same as obtained in the tests.

The determination of MIC to evaluate the susceptibility of planktonic *S. saprophyticus* cells revealed that most samples were susceptible to the antibiotics tested. Regarding oxacillin resistance, 98.3% of the planktonic cells were resistant in the microdilution test, but only three isolates were positive for the *mecA* gene. The three samples that were positive for the *mecA* gene showed the highest MIC (256 μg/mL) and the remaining 116 had MIC ranging from ≤ 0.25 to 2 μg/mL. Similar results have been reported in other studies and might be due to the fact that the breakpoint recommended by the CLSI overestimates resistance in this species (Ferreira et al., 2012).

In general, the antibiotics tested proved to be ineffective in *S. saprophyticus* biofilms as resistant isolates were found for all drugs tested. This is a matter of concern because high doses of antibiotics would be necessary to eliminate these microorganisms organized in biofilms, which is clinically impractical. Biofilm cells may be more resistant to antibiotics because the bacteria are protected against the action of the drugs, with the biofilm impairing the entry of molecules by acting as a physical barrier for diffusion. In addition, biofilm cells have reduced metabolic and growth rates and the biofilm matrix can adsorb or react with the antibiotics, thereby reducing the amount of antibiotics available to interact with cells in the biofilm. Another possibility is that the biofilm cells are tolerant to antibiotics. Hence, treatment may lead to the eradication of most part of the biofilm population, but a fraction of persistent cells is not affected and thus acts as a nucleus for reinfection after therapy discontinuation (Lewis, 2012).

The microorganisms inside a biofilm express different phenotypic characteristics when compared to their free-living homologs. In a study investigating whether the antibiotic resistance genes *aac6-aph2a*, *ermC*, and *tetK*, which confer resistance to gentamicin, erythromycin and tetracycline, are likely to be disseminated via conjugative transfer, Águila-Arcos et al. (2017) searched for horizontal transfer genes from two common staphylococcal plasmids, (i) conjugative pSK41 and (ii) mobilizable pT181, in 25 staphylococcal biofilm-forming clinical isolates belonging to the species *S. aureus*, *S. epidermidis, S. hominis,* and *S. capitis*. Both horizontal transfer and antibiotic resistance genes were detected in these staphylococcal isolates. Therefore, biofilms represent a hot spot for horizontal gene transfer by bacterial conjugation. This horizontal gene transfer is important for the genetic diversity of microbial communities and favors the exchange of genes that can contribute to the chronic nature of infections (Vuotto et al., 2014). The detection of horizontal transfer and antibiotic resistance genes in these clinical staphylococcal strains isolated from biofilms points to the potential risk of the development and dissemination of multidrug-resistant bacteria.

The most commonly prescribed antibiotics for the treatment of UTIs are trimethoprim/sulfamethoxazole, fluoroquinolones (ciprofloxacin or norfloxacin), first and second generations of cephalosporins, amoxicillin + clavulanate, and nitrofurantoin (Lee et al., 2008). In the present study, 17.7% of the samples were already resistant to trimethoprim/sulfamethoxazole in the evaluation of planktonic MIC, while 48.7% of the biofilm samples were resistant. In addition, 31.1% of the samples changed from susceptible to resistant in the biofilm state, an alarming finding considering that the trimethoprim/sulfamethoxazole combination is considered the first-line drug for the treatment of uncomplicated UTIs (Drekonja and Johnson, 2008). Thus, the frequent use of the drug in empirical therapy is associated with an increase in the clinical failure rate, especially if the microorganism grows in biofilms, as observed in the present study.

The administration of fluoroquinolones is recommended for uncomplicated UTIs in areas where the incidence of trimethoprim/sulfamethoxazole resistance is higher than 10%, as well as for the treatment of complicated UTIs and acute pyelonephritis (Blondeau, 2004). Fluoroquinolones have been successfully used to treat a wide range of community-acquired and hospital-acquired infections, and rates of resistance to fluoroquinolones remain low (Oliveira et al., 2016). In fact, in the present study, all planktonic samples were susceptible to norfloxacin and ciprofloxacin, however, the same was not observed for the biofilm samples, with 34.4% of the isolates exhibiting intermediate resistance or resistance to norfloxacin and 25.2% exhibiting intermediate resistance or resistance to ciprofloxacin. The presence of the biofilm increased the MIC by two, four, eight and up to 32 times the values obtained for some drugs, with some samples switching from the category of susceptible in the planktonic condition to resistant in the biofilm state. This phenomenon was more frequently observed for norfloxacin, ciprofloxacin, and trimethoprim/sulfamethoxazole.

Oliveira et al. (2016) evaluated the MIC for planktonic and biofilm cells of *Staphylococcus* spp. comparing six drugs, and observed a two-, four-, eight-, and up to 16-fold increase of MIC in the presence of the biofilm compared to planktonic cells, mainly for the drugs vancomycin and erythromycin. In that study, among the 20 *S. saprophyticus* isolates studied, no planktonic samples were resistant to vancomycin and linezolid. However, regarding the MICB, the percentage of samples that moved from susceptible to resistant or intermediate resistant was 53.8% for vancomycin and 30.8% for erythromycin. The authors also observed that *S. haemolyticus*, *S. hominis*, *S. warneri*, and *S. lugdunensis* isolates did not exhibit much variation of MIC in the presence of the biofilm, probably because these species are poor biofilm producers.

Regarding determination of MBCB in the present study, there were isolates for which the MBCM of all drugs was equal to or higher than the highest concentration tested. The results corroborate the observation that microorganisms susceptible to certain antimicrobials in conventional laboratory tests may be highly resistant to the same antimicrobials when grown in biofilms. Consequently, infectious diseases involving biofilms are generally difficult to treat. Bacterial biofilms play an important role in UTIs, being responsible for persistent infections that lead to recurrences and relapses (Delcaru et al., 2016).

Studies have demonstrated the importance of bacterial biofilm formation in UTIs, particularly chronic cystitis and catheter-associated infections (Hancock et al., 2007). Urinary catheters and other prosthetic devices predispose to UTIs by serving as a substrate for biofilm formation, carrying a higher bacterial burden and increasing the risk of epithelial adhesion.

The finding that *S. saprophyticus* isolates can produce biofilms, in addition to the observation of resistance to the antimicrobial agents when these microorganisms were grown in biofilms, suggests that biofilm formation is a very important virulence factor for *S. saprophyticus*, which permits this species to establish persistent UTIs. This study demonstrated that the severity of UTIs depends not only on the susceptibility of the microorganism to the antibiotics commonly used for treatment, but also on the virulence of the bacteria. Biofilm production by *S. saprophyticus* and its role in UTIs remain poorly studied. Treatment of this infection is usually simple and rapid, however, if not treated correctly with efficient antimicrobials, progression to much more severe infection of the kidneys (pyelonephritis) may occur that can lead to generalized infection, renal abscesses, and loss of kidney function. No data are available correlating the inefficacy of antibiotics in the treatment of UTIs with the biofilm formation by *S. saprophyticus* or any other species. However, the results of the present study show that more attention should be given to this virulence factor in *S. saprophyticus* and to the antimicrobial treatments used since *in vitro* biofilm formation decreases the susceptibility of the microorganisms to the antibiotics tested. The results of conventional antimicrobial susceptibility tests (MIC) cannot be applied to microorganisms grown in biofilms as the antimicrobials tested were unable to eradicate biofilm-bound bacteria. This was clearly demonstrated in the present study.

## Conclusion

The present study shows that biofilm production is a successful strategy for the microbial survival of *S. saprophyticus* and should be taken into account in the treatment of UTIs that do not consistently respond to therapeutic concentrations, as the response to antimicrobials may be impaired in bacterial biofilms. This virulence factor may increase the survival capacity of the pathogen during the treatment of infection with antimicrobial agents.

## Author Contributions

KM participated in the conception and design of the study, carried out the microbiological tests, and wrote the manuscript. AF provided the clinical material and helped with the conception and design of the study. VP, LP, and AO helped with the microbiological tests. MC was responsible for the conception and design of the study, coordination of laboratory work, data analysis, and manuscript writing.

## Conflict of Interest Statement

The authors declare that the research was conducted in the absence of any commercial or financial relationships that could be construed as a potential conflict of interest.
